# Consistency of Network Modules in Resting-State fMRI Connectome Data

**DOI:** 10.1371/journal.pone.0044428

**Published:** 2012-08-31

**Authors:** Malaak N. Moussa, Matthew R. Steen, Paul J. Laurienti, Satoru Hayasaka

**Affiliations:** 1 Neuroscience Program, Wake Forest School of Medicine, Winston-Salem, North Carolina, United States of America; 2 Department of Radiology, Wake Forest School of Medicine, Winston-Salem, North Carolina, United States of America; 3 Department of Biostatistical Sciences, Wake Forest School of Medicine, Winston-Salem, North Carolina, United States of America; Hangzhou Normal University, China

## Abstract

At rest, spontaneous brain activity measured by fMRI is summarized by a number of distinct resting state networks (RSNs) following similar temporal time courses. Such networks have been consistently identified across subjects using spatial ICA (independent component analysis). Moreover, graph theory-based network analyses have also been applied to resting-state fMRI data, identifying similar RSNs, although typically at a coarser spatial resolution. In this work, we examined resting-state fMRI networks from 194 subjects at a voxel-level resolution, and examined the consistency of RSNs across subjects using a metric called scaled inclusivity (SI), which summarizes consistency of modular partitions across networks. Our SI analyses indicated that some RSNs are robust across subjects, comparable to the corresponding RSNs identified by ICA. We also found that some commonly reported RSNs are less consistent across subjects. This is the first direct comparison of RSNs between ICAs and graph-based network analyses at a comparable resolution.

## Introduction

In a typical fMRI data set acquired during resting-state, BOLD (blood-oxygen level-dependent) signals often exhibit strong correlations between distant brain areas despite a lack of external stimuli or a cognitive engagement [1]–[3]. Such elevated correlation, known as functional connectivity, has been identified in the motor cortex [1], the dorsal and ventral pathways [3], and the default mode network (DMN) [4], to name a few. One way to find such networks following similar time courses is ICA (independent component analysis). Without an explicit model, ICA is able to separate time course data into a collection of independent signals, or components, with each component representing a network following a similar temporal pattern. For example, Damoiseaux et al. [5] examined resting-state fMRI data using spatial tensor PICA (probabilistic ICA) and discovered 10 components that consistently occurred in multiple subjects. Similarly, De Luca et al. [6] identified 5 distinct resting-state networks (RSNs) in BOLD fMRI data as 5 ICA components. More recently, Doucet et al. [7] examined the hierarchical structure of 23 components found by ICA and identified 5 major clusters among those. Throughout the text, such a network following a similar temporal pattern discovered by ICA is referred as a “component.”

Another approach to finding temporally correlated areas in resting-fMRI data is a graph theory-based approach. In such an approach, a functional connectivity network can be constructed based on a strong temporal correlation between brain areas [8]. In particular, various brain areas, represented as nodes, are considered connected to each other if the correlation between them is strong. These strong correlations among nodes are represented by edges connecting the nodes. In the resulting graph representing the brain network, some subsets of nodes may be highly interconnected among themselves, effectively forming communities of nodes. Such communities of nodes, also known as modules, have been identified in a number of brain network studies of resting-state fMRI [9]–[13], and although the number of nodes may substantially differ in these studies, the number of modules seems fairly comparable. Such modules represent areas of high temporal coherence in the brain, and some of the modules coincide with the RSNs discovered by ICA. For example, a module corresponding to the default mode network has been reported by multiple studies [9], [11], [13] whereas a module covering the motor network was also found in some studies [9], [10], [12], [13]. However, comparing the network modules directly to RSNs from ICA is challenging due to the difference in their spatial resolutions. While RSNs from ICA have a voxel-level resolution, most whole-brain networks are typically much coarser and consist of only a few hundred nodes. It is worthy to note here that, recently, a study combined the spatial ICA and graph theoretical analysis to demonstrate topological properties of each RSN [14].

Even though both ICA and graph theory-based network approach can find similar organization structure in the brain, a network approach offers two advantages. First, a network approach can be used to assess similarity or differences in overall network structure quantitatively. Recent advances in network science provide methods to examine how network modules change over time [15], [16]. Such techniques have been applied to fMRI data to examine dynamic reconfiguration of brain network organization [17]. Secondly, a network approach can examine how different modules are connected to and interact with each other. Although network modules tend to form cliques of their own, such modules are also connected to other modules, allowing exchange of information and forming the network as a whole. This is in contrast to ICA, in which each component is independent and isolated from the other components. Therefore, when functional brain networks are constructed at the voxel-level, a resolution similar to ICA, a network based approach offers distinct advantages over ICA in understanding the overall organization of the brain network.

A major challenge in examining network module organization is to summarize the consistency of modules across subjects. This is particularly a concern since each subject’s network structure varies slightly from other subjects even though the overall organization appears similar. One possible solution is to generate a group network summarizing the consistent network connectivity observed in a large number of subjects. Examining the modular organization of the resulting group network may enable evaluation of consistent network modules. The notion of an “average” network sounds very appealing in such a scenario. In fact, several functional brain network studies have generated a group network by simply averaging the correlation coefficients between the same set of nodes across subjects [10], [12], [13]. Another study has examined whether or not the correlation coefficient between each voxel pair significantly differs from zero [11]. Although averaging correlation matrices across subjects can represent the connectivity between two nodes as an element in the averaged matrix, such an approach may not accurately summarize the consistent network structure. In other words, such an approach may adequately capture the connection strength between nodes A and B, but this method does not consider how node A is connected to other nodes in the network.

In this work, we attempt to examine modular organization of the resting-state brain network and compare the results to that of the RSNs identified by ICA. To do so, we constructed functional brain networks with fMRI voxels as network nodes [18], and thus the resulting network resolution is comparable to that of previous ICA studies. We then examined network modules in these voxel-based networks for consistency across subjects, and whether consistent modules are comparable to the RSNs found by ICA studies. To do so, we employed scaled inclusivity (SI), a metric quantifying consistency of modules across multiple networks of a similar type [16]. Our hypothesis is that, if RSNs are stable across subjects, our approach should be able to identify such RSNs as network modules associated with high SI. Since SI can be calculated at the nodal level, the consistency of the resulting modules can be assessed at the voxel-level. Moreover, this allows us to compare the consistency of modules to the variability of the corresponding RSNs observed in an ICA study [5].

## Results

The data used in this work were part of the 1,000 Functional Connectomes Project (http://fcon_1000.projects.nitrc.org/), a collection of resting-state fMRI data sets from a number of laboratories around the world. Of all the data sets available, 4 data sets from 4 different sites (Baltimore, Leipzig, Oulu, and St. Louis), consisting of n = 194 subjects in total, were selected because (i) these data sets consisted of young to middle-aged subjects (20–42 years old) and (ii) these data sets were acquired while subjects’ eyes were open and fixated on a cross. The original resting-state fMRI data were processed using the same preprocessing pipeline available in our laboratory (see Materials and Methods). Networks were formed by calculating a correlation coefficient for every voxel pair then by thresholding the resulting correlation matrix to identify strong correlations. The threshold was adjusted for each subject in a way that the density of connections was comparable across subjects (see Materials and Methods). Each voxel was treated as a node in the resulting network. Each subject’s network consisted of an average of 20,743 nodes. Modules in each subject’s network were identified by the Qcut algorithm [19]. The algorithm identified sets of nodes that were highly interconnected among themselves and designated them as distinct modules. Each node in the network can only be part of one module at a time.

**Figure 1:**
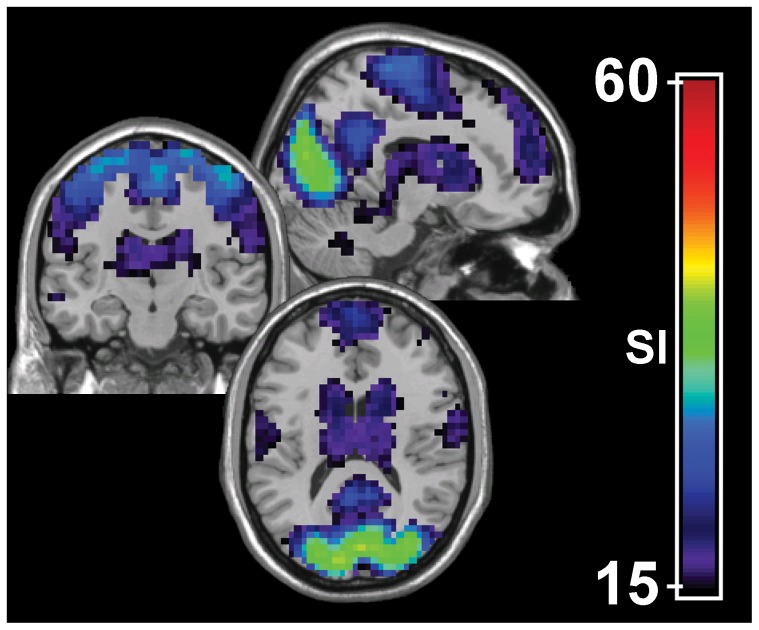
Consistency of whole-brain functional modular organization across subjects. Global scaled inclusivity (SI) shows that several brain regions are consistently partitioned into the same modules across individuals. These areas include portions of the following cortices: visual, motor/sensory, precuneus/posterior cingulate, basal ganglia, and frontal.

After modules were identified in all the subjects, the consistency of modules across subjects was assessed using SI. In brief, SI summarizes the overlap of nodes in modules across different subjects while penalizing any disjunction between modules (see Materials and Methods). SI is calculated at each node, forming an SI image summarizing across-subject consistency of the modular structure. More specifically, each SI value measures how consistently a particular node falls into a particular module. A high SI value indicates that the voxel is located in the same module across subjects, while a low SI value signifies that the voxel is likely part of different modules in different subjects. Theoretically, SI ranges from 0 to n–1 (n–1 = 193 in this study) [16]. However, in practice the SI values are considerably lower than the possible maximum value of n–1 due to disjunction between modules across subjects. Figure 1 shows the SI image generated from all the subjects’ modular organization, thresholded at SI>15. This threshold was maintained throughout the manuscript to facilitate comparison between modular organizations. The areas of high SI correspond to areas that were consistently part of the same modules across subjects. These areas include the occipital lobe, precuneus, posterior cingulate cortex, pre- and post-central gyri, medial frontal gyri and the components of basal ganglia.

During the calculation of the global SI map shown in Figure 1, we were able to determine which subject’s module was the most representative at a particular node (see Materials and Methods) [16]. This representative module resulted in the largest SI value at that particular voxel location among all the subjects’ modules. To further examine high SI areas, the most representative modules that correspond to the brain regions in Figure 1 were identified. These representative modules were then used to summarize consistency among subjects and SI was calculated with respect to these modules. The resulting images are module-specific SI images and summarize group consistency at a voxel-level. Module-specific SI images are analogous to coefficient of variation (CV) images, which are used in ICA analyses to summarize consistency of RSNs at the voxel-level [5].

**Figure 2:**
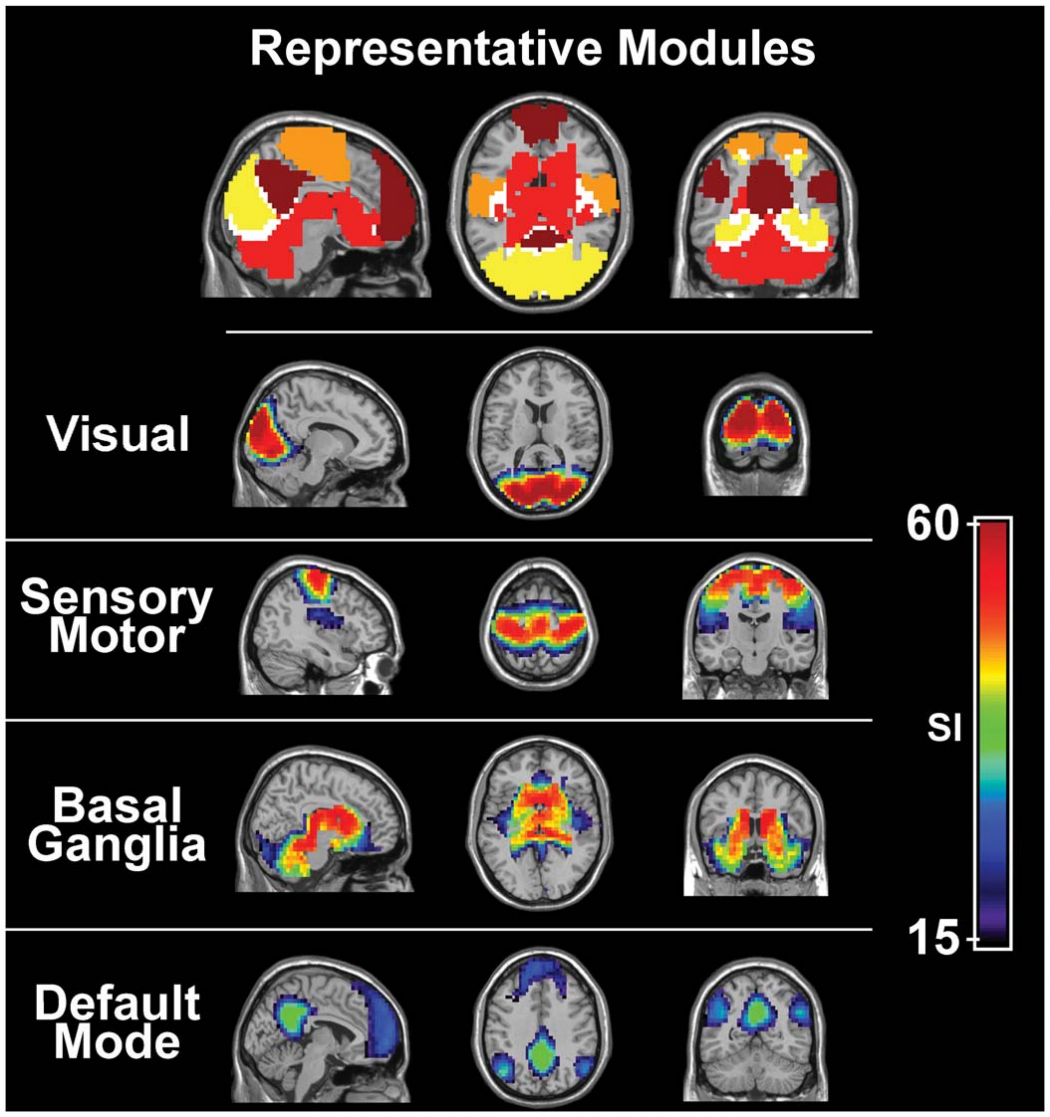
Module-specific SI of four most consistent modules across subjects. *Row 1*: Four functional modules were found to be highly consistent across subjects. These modules include the visual (yellow), sensory/motor (orange) and basal ganglia (red) cortices as well as the default mode network (precuneus/posterior cingulate, inferior parietal lobes, and medial frontal gyrus; maroon). Overlap among these modules was present but minimal (white). *Rows 2–5*: Module-specific SI images for each of the four most consistent modules, namely the visual (row 2), sensory/motor (row 3), basal ganglia (row 4), and default mode (row 5) modules. Note that the visual, sensory/motor and basal ganglia all show higher consistency across subjects than the default mode module. Among the default mode areas, the precuneus and posterior cingulate cortex show the greatest consistency across subjects.

The visual module covers the entire span of visual cortex and includes both primary and secondary cortices (Figure 2). This module is comparable to ICA components like components A and E in Damoiseaux et al. [5], RSN1 in De Luca et al. [6], and module M2b in Doucet et al. [7]. The corresponding module has also been reported in previous functional brain network analyses, including Module II of He et al. [11], Module 4 of Rubinov and Sporns [12], and the posterior module of Meunier et al. [10]. Thus, this module is highly consistent among individuals and easily identifiable by both ICA and network methodologies. Moreover, the secondary cortices of the occipital lobe exhibited high SI values (Figure 2), which is comparable to the reduced variability observed in visual components found by a previous ICA study [5].

The sensory/motor module (Figure 2) is analogous to the motor network identified by the seed-based correlation method [1]. The most consistent regions within this module include the pre- and post-central gyri. On the other hand, the supplementary somatosensory area (S2), surrounding auditory cortex and portions of the posterior insula show reduced consistency across subjects. This module roughly corresponds to component F in Damoiseaux et al. [5], RSN3 in De Luca et al. [6], and module M2a in Doucet et al. [7]. Similar to the results reported by Damoiseaux et al. [5], the consistency of this module was lower than that observed for both default mode network (DMN) and visual modules (Figure 2). Module I of He et al. [11] and Module 1 of Rubinov and Sporns [12] demonstrate similarities with our sensory/motor module. Interestingly, these previously reported modules also include portions of the insula and auditory cortices. These findings are not only consistent with ours but also to previous reports of the ICA results.

The basal ganglia module (Figure 2) consisted of the caudate, globus pallidus, putamen, and thalamus. It also extended into the medial temporal lobe, temporal pole, parahippocampal gyrus, hippocampus, amygdala and cerebellum. Interestingly, these brain regions have not been consistently classified into one component by ICA. While De Luca et al.’s RSN3 suggests some involvement of the hippocampus and thalamus [6] within the motor component, some ICA studies did not find a component similar to this module [5], [7]. However, another ICA study by Damoiseaux et al. revealed a component consisting of the thalamus, putamen and insula (component K) [20] which led to other ICA studies on connectivity. In particular, the basal ganglia component has been shown to include portions of the striatum, such as the caudate and the globus pallidus [21]–[23]. Similarly, basal ganglia modules have been previously reported in studies that have used network methodologies. For example, Module V found by He et al. [11] and Module 3 by Rubinov and Sporns [12] contain all the regions of the basal ganglia. Variations of this have also been described in the central module of Meunier et al. [10] and in the RSN3 of De Luca et al. [6]. Though these findings contain similar regions as our module, they extend further into the insular and motor cortices. Functional connectivity of the cerebellum with the rest of the basal ganglia proved unique in our results compared to previous network module findings. Although global SI (Figure 1) values did not indicate high modular consistency of the cerebellum across subjects, the module-specific SI map shows that it is consistently part of the basal ganglia module across subjects (Figure 2).

The default mode network (DMN) [4], [24] was also identified as a consistent module across subjects (Figure 2). This module included the precuneus (PCun), posterior cingulate cortex (PCC), inferior parietal cortex, superior medial frontal cortex, and anterior cingulate cortex (ACC). The PCC exhibited elevated SI values and was found to be the most consistent brain region of the DMN. In comparison, the SI values of the medial frontal gyri were attenuated, indicating this region to be less consistently found in the DMN module.

The intra-modular consistency of this module appeared comparable to the reduced variability of the DMN component found by an ICA [5]. While this module covers the brain areas typically considered as part of the DMN, weaker SI in the frontal portion also suggests that the anterior and posterior portion of the DMN may not be as strongly coupled as the rest of the DMN. This may be because the connectivity pattern is slightly different between the anterior and the posterior portions of the DMN. Research supporting this hypothesis includes that of Andrews-Hanna et al. [25] using temporal correlation analysis. They determined that the DMN was composed of multiple components, including a medial core and a medial temporal lobe subsystem. Using ICA, Damoiseaux et al. [20] described two RSN components that together included the superior and middle frontal gyrus, posterior cingulate, middle temporal gyrus and superior parietal cortices. Finally, the work of Greicius et al. notes some differences in the seed-based connectivity of the DMN when the seed was placed in either the PCC or the ventral ACC [2].

**Figure 3:**
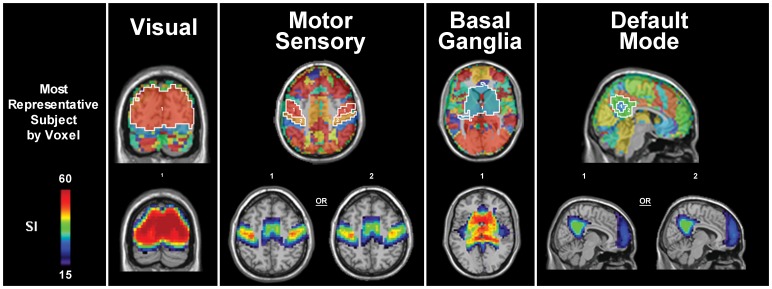
Multiple representative individuals produce similar module-specific SI maps. Of the four functional modules that were found to be highly consistent across subjects, two (motor/sensory cortices and the default mode) had multiple representative subjects that could have been chosen to calculate module-specific SI. Here we show that in each case the resulting module specific SI map is similar in the brain areas that are included as part of the overall module. For instance, images of the most representative subject by voxel location (*top panel*) show that two individuals are the most representative for the motor and sensory cortices, respectively. However, when each of these individuals was used to calculate module-specific SI it was found that the resulting module included both cortices.

Among the modules shown in Figure 2, there were more than one choice for the most representative subject in the sensory/motor module and the default mode module. This can be seen in Figure 3 showing the image of the most representative subject by voxel locations. Within the motor / sensory strip and the precuneus, there were two subjects with the highest SI values. Even though either of these subjects could serve as the representative subject for these modules, the overall consistency of the entire module was still captured, as the module specific SI images appear strikingly similar even if different subjects were chosen as the representative subject (Figure 3).

**Figure 4:**
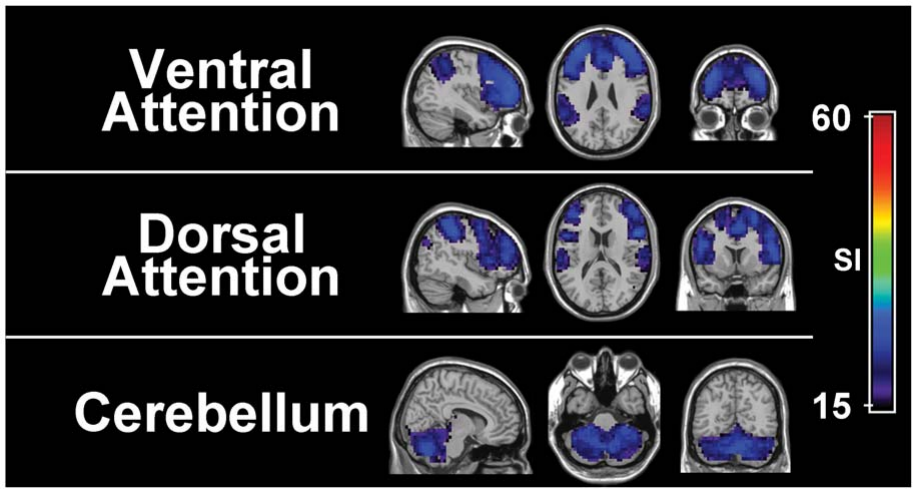
Module-specific SI images of modules with limited consistency. Three resting state networks (RSNs) exhibited attenuated consistency across subjects, relative to those shown in Figure 2. Module-specific SI images are shown for the ventral attention network (superior parietal lobules, dorsal lateral prefrontal cortex and portions of the medial frontal gyrus, row 1), dorsal attention network (superior parietal lobules, intraparietal sulci, precentral and superior frontal gyri, row 2), and the cerebellum module (row 3).

The number of modules in Figure 1 seems surprisingly few, especially when compared to previous reports of ICA [5], [7]. Our results, however, do not indicate the absence of modules similar to previously found ICA components. Instead, some were only found to be less consistently organized across subjects (Figure 4). These modules do not necessarily include similar sets of nodes across subjects, and consequently do not exhibit high global SI values (Figure 1). Two of such modules are the ventral (superior parietal cortex as well as superior and medial frontal gyri) and dorsal (superior parietal cortex, superior and dorsal lateral frontal, and precentral gyri) attention networks identified by previous fMRI analyses [3]. A previous ICA finding has combined these two systems into the same component [6] while others have separated them into separate components for the left and right hemispheres [5], [7]. Here we present two distinct modules corresponding to the separate ventral and dorsal attention systems which have also been found in previous network analyses [11], [13]. It is interesting to note that low SI values in our ventral and dorsal attention modules (Figure 4) are in contrast to the stability of corresponding components found using ICA [5].

In addition to the ventral and dorsal attention modules, we present a module containing the cerebellum (Figure 4). Though the cerebellum was found to be consistently connected to the basal ganglia (Figure 2), many nodes within the cerebellum formed a unique module by themselves. However, reduced module-specific SI values indicate that this module demonstrates limited consistency across subjects. Thus, while the cerebellum may belong to the same module as the basal ganglia in some subjects, in another group of individuals the cerebellum belong to an isolated module as shown in Figure 4.

**Figure 5:**
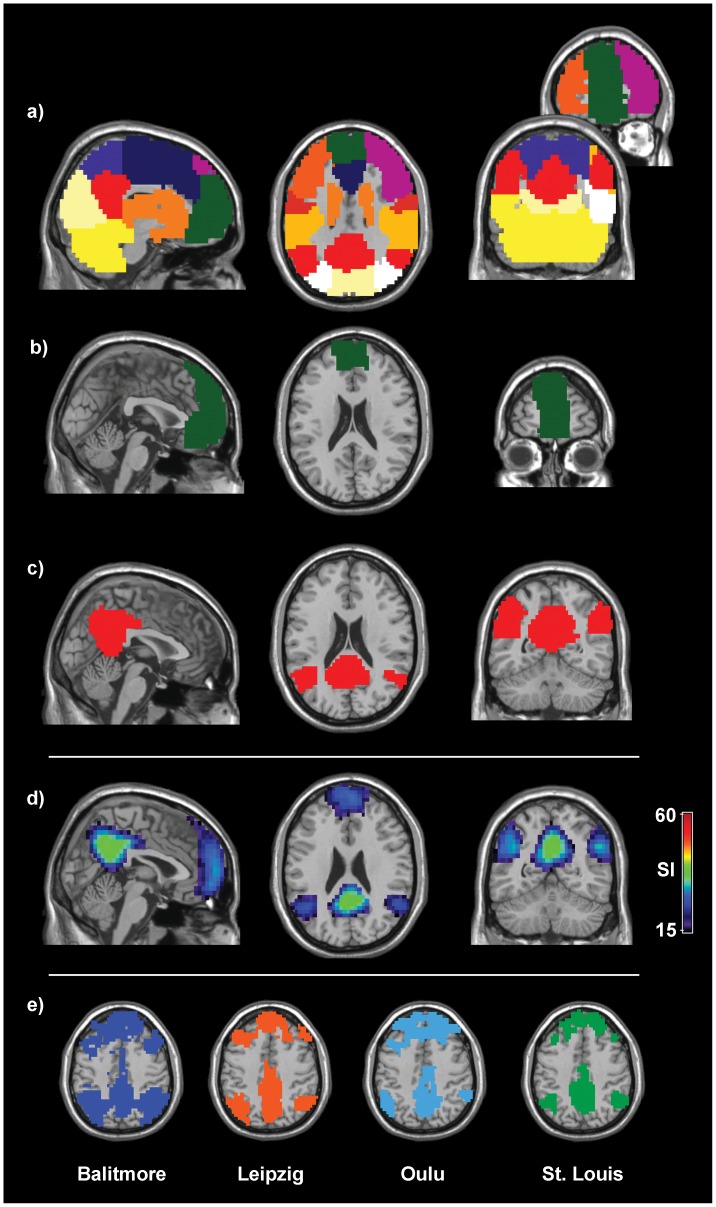
The modular structure of the average network. *a)* The modular structure of the average network, with each color indicating a distinct module. Note that the default mode network is split into two modules: *b)* anterior (medial frontal gyrus, green) and *c)* posterior (precuneus/posterior cingulate & inferior parietal lobes, red) modules. *d)* On the other hand, both anterior and posterior default mode regions appear consistent across subjects when analyzed using module-specific SI. *e)* Representative subjects from each of the four data sets confirm that both anterior and posterior portions of the DMN constitute one module at the individual level.

We used SI to assess the consistency of modules across subjects rather than calculating the average network, which has been used by some researchers to generate a “summary” network for a study population [10]–[13]. An average network, which is produced by averaging correlation matrices across subjects, does not properly represent the characteristics of the individual networks [26]. Rather, it produces a network whose key modular structure is altered from that of the individual networks. Figure 5 shows an example of such an alteration. In particular, we generated an average network by averaging the correlation matrices from all the subjects (n = 194). This average correlation matrix was then thresholded (see Materials and Methods) and modular organization was then detected on the resulting adjacency matrix. The modular organization of this average network is shown in Figure 5a, with each color denoting a network module. The data used in our analysis represent a subset of the subjects used by Zuo et al. [27] and show that modular organization is very similar to theirs. Most striking, however, is the modules associated with the DMN. Using an average network, we found that two distinct anterior (Figure 5b) and posterior (Figure 5c) modules exist. This is in stark contrast to the DMN module-specific SI image, which does not separate into anterior and posterior parts (Figure 5d). To add further confidence in this finding, DMN modules of the individuals of each data set were examined. We found that the anterior and posterior portions of the DMN were indeed commonly found as one module (Figure 5e).

**Figure 6:**
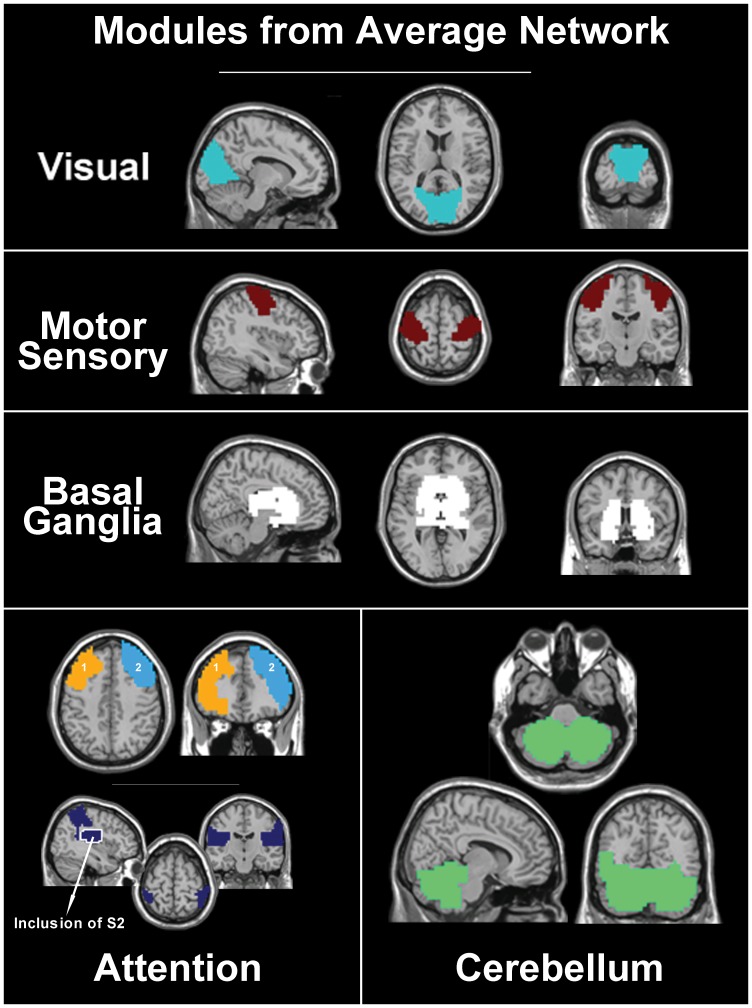
Selected modules from the average network. Shown here are the modules from the average network that correspond to the module-specific SI images shown in Figures 2 & 4. The modules from the average network that correspond to the motor/ sensory cortices, the basal ganglia and the cerebellum were found to be similar with respect to their corresponding module-specific SI image. However, two distinctions were found in addition to those demonstrated in Figure 5. First, the average visual module includes only the area of the primary visual cortex. This is in contrast to the module-specific SI image for the visual cortex shown in Figure 2, which extends into secondary visual cortices. Second, the average network segregates the anterior from the posterior portions of the ventral and dorsal attention systems. In this case, the anterior portion consists of two modules, one for each of the bilateral dorsal lateral prefrontal cortices. Interestingly the posterior element of both ventral and dorsal attention systems (superior parietal lobules) is not separated into bilateral portions. It does, however, include secondary sensory cortices S2.

A comparison between the other three SI modules shown in Figure 2 and the two shown in Figure 4 with those from the average network are presented in Figure 6. Here we show the modules for the visual and motor/sensory cortices as well as the basal ganglia from the average network. These three modules comprise similar areas represented in the module specific SI images of the corresponding modules in Figure 2. In addition to previously mentioned differences (Figure 5b, c), we show that average modules corresponding to the ventral and dorsal attention brain regions are quite different than those found using module specific SI in Figure 4. For instance, averaging correlation matrices across individual subjects resulted in the separation of the left from the right dorsal lateral prefrontal cortex. Neither of these modules included the superior portions of the parietal lobules. Instead, these brain areas were identified as a separate module. Interestingly, this module included bilateral secondary sensory cortices.

**Figure 7:**
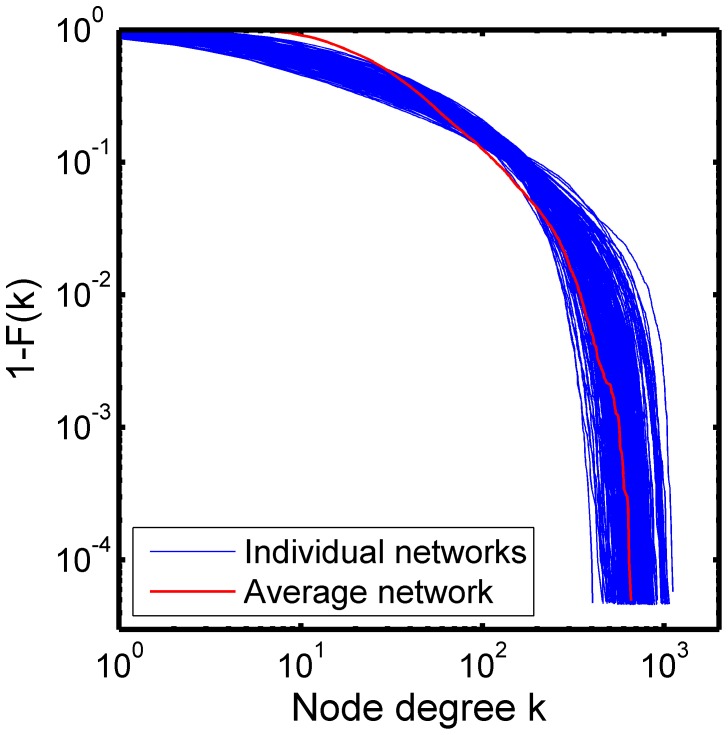
Degree distributions of the average network and individual networks. The distribution of the number of connections at each node, or degree, is plotted for each of the 194 subjects (blue), as well as for the average network (red). The Y-axis is the complimentary cumulative distribution (i.e., 1 minus the cumulative distribution function (CDF)). The average network has more low degree nodes than any of the other individual networks. The degree distribution of the average network, however, drops dramatically for degrees greater than 10, suggesting that there are fewer medium degree nodes.

Averaging alters not only modular organization, but also other network characteristics [26]. Figure 7 shows the distributions of node degree, or the number of edges per node, for all n = 194 subjects (blue) as well as that of the average network (red). As it can be seen in Figure 7, the average network has far more low degree nodes than any of the subjects in the data set. However, the average network lacks medium degree nodes and thus its degree distribution drops faster than that of the other individual networks. Various network metrics are also altered in the average network. For example, the clustering coefficient and the path length, describing tight local interconnections and efficient global communication respectively [28], are significantly different (p<0.0001, one-sample T-test) from that of the individual networks (see Table 1). Taking all these observations together, we can conclude that the average network does not accurately represent characteristics of individual networks in the data.

**Table 1:** Comparison of network characteristics between the average network and individual networks.

	Clustering coefficient C	Path length L
Average network	0.457	7.64
Mean (SD) ofindividual networks	0.353 (0.031)	5.14 (0.52)

## Discussion

In this work, consistency of modules in resting-state functional connectivity networks was examined at the voxel-level, a resolution comparable to that of group ICA. Module consistency across subjects (n = 194) was assessed and the results were compared to ICA components and network modules previously reported by other studies. Modular consistency was assessed using SI, which quantifies inter-subject variability in modular organization. The use of SI also allowed us to examine inter-subject consistency of a particular module at the voxel-level. This showed what brain regions within a module were consistent in an analogous fashion to group ICA results [5]. Our global SI data show that only a handful of brain areas were consistently organized in modules. These modules alone, however, were not found to constitute the entire network. Instead, we show that other network modules are less consistent across subjects; multiple examples are presented to convey this point.

Interestingly, despite the large number of nodes in our brain network data, the number of major modules did not change dramatically from the previously reported brain network modularity [9]–[13], [29]. Increasing the network resolution (the number of nodes used to model the brain as a network) did not result in more modules. Power et al. [13] also discovered a similar number of modules despite differences in the network resolution. This is particularly interesting because Power et al. [13] cautioned against using network nodes that were not derived based on brain functional anatomy. Based on our finding and Power et al.’s finding, we conclude that modular structure is robust and can be ascertained despite differences in the parcellation scheme of the brain. However, a voxel-based network is advantageous since the shape of each module can be determined at finer granularity. A voxel-based network also enables examination of intra-modular characteristics within a particular brain area.

Some RSNs, although reported in multiple studies, were not found to be consistent in our analysis. This may falsely suggest that there are only a handful of modules in the resting-state functional brain network. Other modules, however, exist and are found when modules from each subject’s network are examined carefully. A few examples of such network modules are shown in Figure 4, with somewhat attenuated SI values than the RSN modules reported in Figure 2. Thus, the global SI image needs to be interpreted carefully. It cannot be used to identify a “significant” module that exceeds a certain threshold, an approach commonly used in a typical fMRI analysis. It only enables assessment of modular consistency across subjects and does not eliminate the need to qualitatively evaluate network structure [30]. In fact, the extension of the basal ganglia module into the cerebellum (Figure 2) could not have been observed if this module were not carefully examined.

The work of Kiviniemi et al. [23] serves as a prime example of ICA of data similar in demographic characteristics and scanning protocol to the data in our analysis. Their data, which were collected at Oulu University — one of the sites for the data used in our analysis — identified several components that are similar to modules described in our study. Kiviniemi et al. used peri-Sylvian, occipito-parietal, frontal and temporal signal sources to describe 42 RSN components [23]. These results do bear similarity with our presented findings. For example, they described components consistent with the functional association of major cortical areas, including the visual, sensory and motor cortices. In addition to this, they present a component similar to the dorsal attention module presented in Fig. 4. However, modular analysis of consistent functional neighborhoods in the brain does in fact differ from the results of ICA. The most prominent dissimilarity is the number of components in relation to the number of modules. For instance, the visual module identified in our study corresponds to seven separate components found in Kiviniemi et al. [23]. Also, the ventral attention module described in our results comprised of the DLPFC (dorsolateral prefrontal cortex) and the superior parietal lobules. Using ICA, however, the DLFPC was found to be an isolated component. Finally, Kiviniemi et al. showed that, depending on the number of components, the anterior and posterior portions of the DMN are separated into distinct components [23].

When examining the consistency of modular organization across a group of subjects, one may be tempted to generate an average network and examine its modular organization. This approach seems intuitive and reasonable especially for those neuroimaging researchers who are accustomed to voxel-based analyses of neuroimaging data. The notion of average images may sound reasonable in fMRI analyses examining activation patterns through the averaging of multiple individual activation maps, hence one may believe that averaging connection strengths across subjects may also result in a network that summarizes the overall characteristics of the group. Although such an averaging process may be able to summarize the correlation between two particular nodes, it alters the characteristics of the network as a whole tremendously. Such altered characteristics include the modular organization (Figure 5), degree distribution (Figure 7), and network metrics (Table 1). Moussa et al. also demonstrated that average metrics do not imply regional consistency [31]. Since the average network does not necessarily represent the characteristics of the networks it aims to represent, an alternative approach should be considered in summarizing a collection of networks. For the modular organization in particular, selecting a representative subject, based on the Jaccard index, is a simple solution [9], [32]. The SI-based approach, as used in this paper, is a more sophisticated way to examine consistency of modular organizations across subjects. Several network science methods have been developed to compare the modular organization across multiple networks [15], [16], thus application of such methods in brain network data is more appropriate than simply averaging correlation matrices.

Our use of SI demonstrated consistency of the network modular structure quantitatively. However, there are some limitations associated with our approach. First, in the algorithm we used to identify modules [19], each node can only be part of one module. However, it is plausible that some parts of the brain, in particular multi-modal areas, may be associated with multiple modules at once. In recent years, a number of algorithms have been proposed to analyze overlapping modules [33]–[35] in which some nodes are assigned to multiple modules. Such an algorithm has been applied to an analysis of a 90-node structural brain network and overlap between modules has been outlined [29]. However, interpretation of such overlapping modules is unclear. Moreover, since overlapping module algorithms tend to be computationally intensive, applying such methods to brain networks at the voxel-level may pose a significant challenge. However, the evaluation of modular consistency across a group of individuals can identify multiple modular structures that contain a single brain region. This was observed with the cerebellum in our work. Another limitation of our approach is that the algorithm to identify modules is imprecise. Identifying the true modular structure of a network is an NP-hard problem [36]. Most algorithms that find modular organization, including Qcut [19], can yield only an approximation to the true solution and have some variability associated with each approximated solution. To overcome this problem, we ran Qcut 10 times for each subject’s network, and selected the most representative modular partition as the best solution (see Materials and Methods). Even then, the variability in the modular organization cannot be completely eliminated. However, we believe that, if the modular organization of the brain network is truly robust across subjects, our global SI image can identify nodes that belong to the same module despite some variability in modular partitions. Finally, some issues remain as inherent confounds. One example includes the effect of head movement correction on our analyses and their functional interpretation. For instance, the work of Van Dijk et al. [37] demonstrates the difficulty of controlling for head movement even after extensive correction. This confound has also been described in the work of Power et al. [38] and Satterthwaite et al. [39].

In summary, we found that the functional brain network at resting-state consisted of several modules that are highly consistent across subjects. These modules were analogous to the RSNs found in previous ICA and network analyses, even at the voxel-level resolution. Consistency of these modules across multiple study sites, with different MRI scanners and imaging protocols, indicates robust yet consistent organization of the functional connectivity network at rest. The methodology used in this work can be further extended to examine alterations in the modular structure of the brain network under various cognitive states or neurological conditions.

## Materials and Methods

### Data

Data used in this work is publicly available as part of the 1,000 Functional Connectome Project (http://fcon_1000.projects.nitrc.org/), a collection of resting-state fMRI data sets from a number of laboratories around the world. From all the data sets available, 4 data sets from 4 different sites were chosen, all consisting of young to middle aged subjects (ages 20–42 years old). Namely, Leipzig data (n = 37, male/female = 16/21), Baltimore data (n = 23, m/f = 8/15), Oulu data (n = 103, m/f = 37/66), and St. Louis data (n = 31, m/f = 14/17). BOLD fMRI data in total of n = 194 subjects (m/f = 75/119) were included in our analysis, and all the images were acquired during resting-state with eyes open with a fixation cross.

### Network Formation

The resting-state fMRI time series data from each subject was realigned to the accompanying T1-weighted structural image and spatially normalized to the MNI (Montréal Neurological Institute) template by the FSL software package (FMRIB; Oxford, UK), and any non-brain voxels were removed from the fMRI data. The normalized fMRI data was masked so that only the gray matter voxels corresponding to the areas specified by the AAL (Automated Anatomical Labeling) atlas [40] were included in the subsequent analyses. A band-pass filter (0.009–0.08 Hz) was applied to the masked time series data to filter out any physiological noises and low-frequency drift [18], [41], [42]. From the filtered data, confounding signals were regressed out, including 6 rigid-body transformation parameters generated during the realignment process and 3 global mean time courses (whole-brain, white matter, and ventricles) [18], [41], [42]. Then a cross-correlation matrix was calculated, correlating each voxel’s time course to all other voxels in the data set. The resulting correlation matrix was thresholded with a positive threshold, yielding a binary adjacency matrix describing a network with each voxel as a node. In the adjacency matrix, 0 or 1 indicated the absence or presence of an edge between two nodes, respectively. The threshold was determined in a way that the number of nodes N and the average degree K followed the relationship N = K^2^.^5^. This thresholding method was used in order to match the edge density across subjects [18]. The resulting network had the edge density comparable to other types of self-organized networks of similar sizes [43]. N and K varied among subjects; the averages of N and K were 20,743 (range = 17,255–21,813) and 55.5 (range = 53.2–65.5), respectively.

### Module Identification

In a network, the modular organization of nodes can be identified by finding densely connected groups of nodes that are only sparsely connected to other groups of nodes [44]. Thus a network can be partitioned into such groups of nodes, or modules, based on connectivity patterns. There are a number of community detection algorithms, calculating a metric known as modularity Q, a quality function describing optimal modular partition [44]. Finding the optimal community structure, or maximizing Q, is an NP-hard problem [36]. Thus most algorithms only find an approximate modular partition of a network, and such algorithms often produce a different solution for each run. In this work, we used an algorithm called Qcut [19] to find modular organization in each subject’s brain network. Since Qcut is an algorithm producing a different solution in each run, it was run 10 times for each subject’s network, and the solution producing the highest Q was selected as the representative modular partition for that subject. The number of modules varied across subjects, with 14.5 modules in each subject’s network on average (range = 6–29).

### Global Scaled Inclusivity

**Figure 8:**
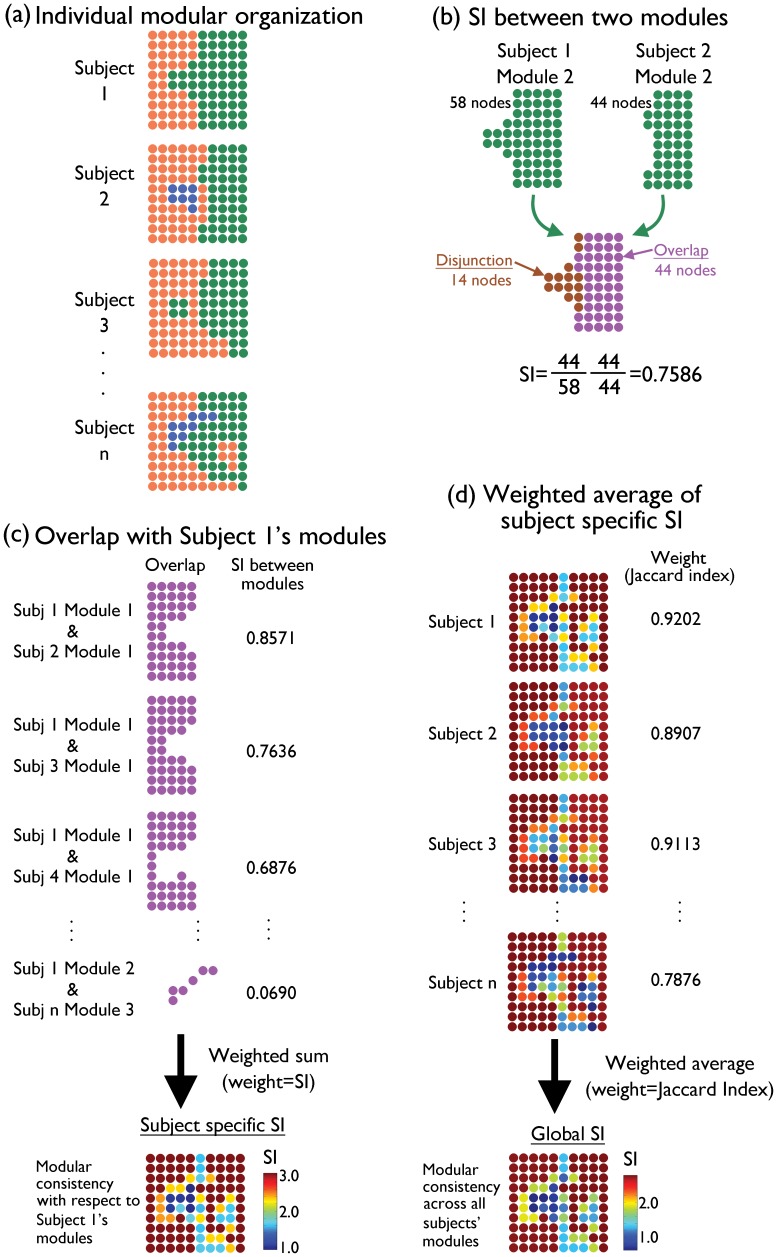
A schematic of global SI calculation. Although the modular organization appears similar across subjects, modules slightly vary from subject to subject (a). Different colors denote nodes belonging to different modules. Among the subjects, one subject is chosen as the referent subject, and any overlap between that subject’s modules and any other modules from the other subjects are determined (b). This process results in maps of overlapping nodes between modules, along with SI values summarizing the fidelity of the overlaps. A weighted sum of the overlap maps, with the SI values as the weights, is calculated, yielding a subject-specific SI map (c). A weighted average of the subject-specific SI maps, with the Jaccard indices as weights, is then calculated, resulting in the global SI map summarizing the consistency of the modular organization across subjects at the nodal level (d).

Scaled inclusivity (SI) was developed as a metric to evaluate consistency of the modular organization across multiple realizations of similar networks. It is calculated by measuring the overlap of modules across multiple networks while penalizing for disjunction of modules. For example, a node V is part of module A in subject i and module B subject j. Then SI for node V, denoted as SI_V_, is calculated as
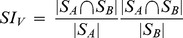
(1)where S_A_ and S_B_ denote sets of nodes in modules A and B, respectively, and |⋅| denotes the cardinality of a set [16]. Figure 8 shows a schematic of how SI can be calculated across different subjects. Although the overall modular organization is similar across subjects, modules slightly vary from subject to subject (Figure 8a). To assess the similarity between two modules from two different subjects, SI can be calculated based on (1) (see Figure 8b). If the two modules A and B consist of the identical set of nodes, then SI_V_  = 1. As the overlap between S_A_ and S_B_ diminishes, the numerator of (1) decreases, leading to SI_V_<1. Or, if either S_A_ or S_B_ is larger than the other, then the denominator of (1) increases, resulting in SI_V_<1.

SI can be calculated between all modules in a particular subject, or the referent subject, against modules from all the other subjects [16]. If there is any overlap between the referent subject’s module and a module from another subject, then SI is calculated between the modules and the overlapping nodes are identified (see Figure 8c). This process results in maps of overlapping nodes between the referent subject’s modules and the other subjects’ module, with the corresponding SI values (Figure 8c). A weighted sum of these maps is calculated, using SI as the weight, and the result is a subject-specific SI map. The subject-specific SI map shows the consistency of the referent subject’s modules when compared to the modular organization of all the other subjects (Figure 8c). In the subject-specific SI map, each node’s SI value reflects how consistently that particular node falls into the same module across subjects. Although a subject specific SI map can summarize the consistency of the modular organization across subjects, it is highly influenced by the choice of the referent subject [16], as can be seen in Figure 8d. In order to avoid a potential bias caused by selection of a particular referent subject, subject-specific SI maps from all the subjects are summarized as a weighted average, with the Jaccard index for each subject as the weight. The Jaccard index summarizes the similarity in modular partitions between two subjects as a single number, ranging from 0 (dissimilar) to 1 (identical) [19]. The Jaccard indices are calculated between each subject against all the other subjects, and the resulting indices are averaged. The average Jaccard index for each subject describes how similar that subject’s modular partition is to all the other subjects’. The average Jaccard indices are appropriately scaled during the weighted averaging process. The resulting weighted average map is the global SI map, demonstrating the consistency of modules at each node (see Figure 8d).

The group SI image is scaled between 0 and n–1; if SI = n–1 at a particular node, that means that node is in the same module with exactly the same set of nodes in all the subjects. Needless to say, such an occurrence is very rare in the brain network. Details on the calculation of the global SI is found in Steen et al. [16]. In order to calculate SI across subjects, it is imperative that all the subjects’ networks have the same set of nodes. Since some subjects’ networks had fewer nodes than that of the others, artificial isolated nodes were also included to match the number of nodes. These artificial nodes were treated as a single dummy module during the calculation of SI, and later eliminated from the group SI image.

### Module-Specific Scaled Inclusivity

**Figure 9:**
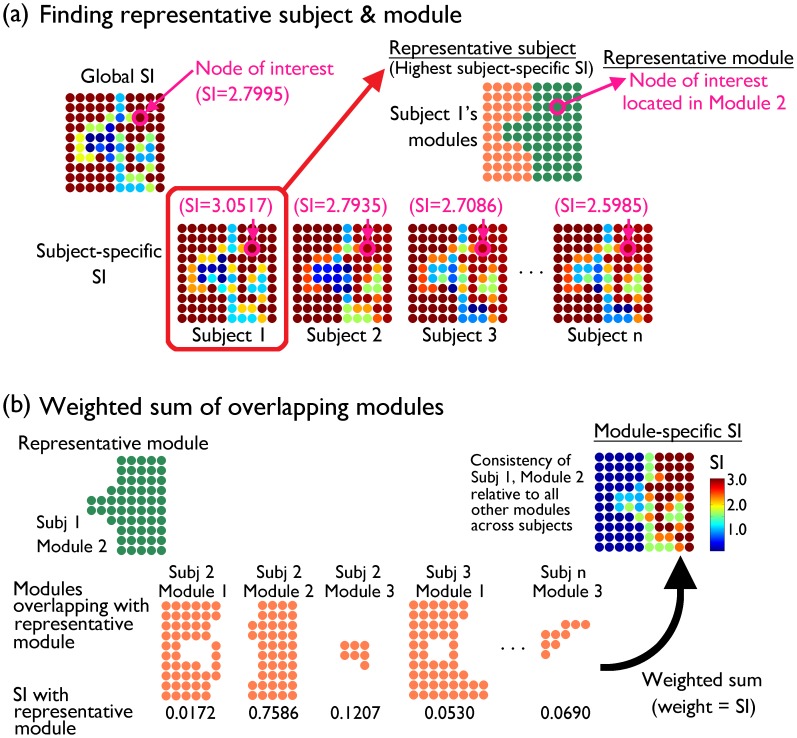
A schematic of module-specific SI calculation. For a particular node of interest, the most representative subject with the highest SI is determined from subject-specific SI maps (a). Then the modular organization of the representative subject’s network is examined, and the module containing the node of interest is identified as the representative module. Next, modules with any overlap with the representative module are identified, and the corresponding SI values are calculated (b). A weighted sum of the overlapping modules is calculated with the SI values as weights, summing modules centered around the representative module. The resulting module-specific SI shows the consistency of the representative module across subjects.

As described above, the global SI image is calculated based on multiple subject-specific SI images (see Figure 8). Consequently, at a particular node location, it is possible to determine the subject yielding the highest SI value, referred as the representative subject (Figure 9a). The highest SI value at that particular node location indicates that the module from the representative subject is considered most consistent across subjects. It is possible to visualize which subject is most representative at different voxel locations, as seen in Figure 3. It should be noted that representative subjects represented in Figure 3 exhibit some spatially consistent pattern, indicating that the most representative subject at one voxel location is likely the most representative subject in the neighboring voxels as well. Once the representative subject is identified, its modular organization is examined and the module containing the node of interest is identified (Figure 9a). That module is considered as the representative module yielding the highest SI at that particular node location.

Once the representative module is identified in the representative subject’s network, then it is possible to evaluate SI between that particular module and modules from all the other subjects. Modules with any overlap with the representative module are recorded, along with the corresponding SI value (Figure 9b). All nodes in the overlapping modules, not just overlapping nodes, are recorded during this process; this is in contrast to the global SI calculation (Figure 8d) in which only the overlapping nodes are recorded. Finally a weighted sum of the modules is calculated, with SI values as weights, resulting in the module-specific SI map (Figure 9b). Such a module-specific SI map shows the consistency of the representative module across subjects. This is because a module-specific SI map summarizes any modules centered around the representative module by summing them together. Although nodes belonging to the representative module may have high SI values, nodes outside the representative module can also have high SI values if those nodes are consistently part of the same module across subjects [16]. A module-specific SI image has the same range as the global SI image, from 0 to n–1. As in the global SI image, SI = n–1 means all the subjects had exactly the same module comprising exactly the same set of nodes.

### Average Network

In brain network analyses involving networks from multiple subjects, some researchers generate an average network in order to summarize the common network characteristics present among the study subjects [10], [11], [13], [27]. However, it is not clear if such an average network truly captures the characteristics of individual networks it aims to represent. In particular, it is not clear whether the modular organization of the network is preserved in an average network. Thus, in order to examine whether an average network has similar characteristics as individual networks, we generated an average network for the data we used in this study. This was done by averaging the correlation matrices from all the subjects, element by element. Since the number of voxels differed across subjects as described above, for each element in the correlation matrix, some subjects may have a valid correlation coefficient for the corresponding node-pair whereas the other subjects may not have a valid correlation coefficient because either node in the node-pair is missing. Thus, in the calculation of the average correlation matrix, the denominator was adjusted for the number of all valid correlation coefficients at each element of the matrix. The resulting correlation matrix was thresholded in the same way as described above, producing an adjacency matrix based on the average correlation. The modular organization of this average network was examined by the Qcut algorithm as described above.
